# Evaluation of electrophotobiomodulation as a recent tool in the armamentarium of treatment of fingertip injuries

**DOI:** 10.1186/s12893-024-02589-8

**Published:** 2024-10-16

**Authors:** Nader Gomaa Elmelegy, Mohamed Saad Sadaka

**Affiliations:** Department of Plastic and Reconstructive Surgery, Faculty of Medicine, Tanta University, Tanta, Egypt

**Keywords:** Electrophotobiomodulation, Fingertip, Treatment

## Abstract

**Background:**

Traditional reconstructive options of fingertip injuries are technically difficult, usually need donor site skin grafting, leave visible scars, and need a protracted period of finger immobilization resulting in joint stiffness. Electro-photobiomodulation (EPBM), is the process of combining intense pulsed light and radiofrequency to modify tissues to help the body heal itself, lower inflammation, and promote wound healing.

**Patients and methods:**

This study included 60 patients presented with fingertip injuries. Patients were randomly divided into two groups. Group 1 includes patients who were treated by EPBM. Group 2 includes patients who were treated by cross finger flap (CFF). Six months after complete healing, evaluation was performed for aesthetic and functional outcome and patient satisfaction.

**Results:**

Compared to group 2 patients, group 1 patients had statistically significant better sensory outcome, better total active motion of affected digits, grip strength, patient satisfaction, healing time, and plastic surgeon general aesthetic evaluation and also, they had statistically significant less adverse events and cold intolerance with absent donor site pain and deformity.

**Conclusion:**

EPBM is safe and effective treatment of fingertip injuries which shortens the healing time, produces the best aesthetic and functional result while avoiding donor site morbidity of the traditional reconstructive options.

## Introduction

The fingertip, a specialized structure that contributes to hand dexterity and responsiveness, is located on the distal phalanx, distal to the point where the flexor and extensor tendons are inserted. More than half of the fingertip’s volume is formed by the volar pulp [1]. Lesions on the fingers are exceedingly common and are responsible for the majority of hand trauma cases that come into the emergency room. Skilled functions of the hand can be significantly impaired due to improper management [2].

The primary objectives of fingertip reconstruction are quick healing, a brief period of functional impairment, and the restoration or reproduction of a sensitive, pain-free fingertip in a completely mobile finger of the maximal feasible length. Skin grafting, primary closure, secondary intention healing, and local or regional soft tissue flaps are among the available treatment options for these injuries. Cross finger flap is one of the commonest workhorse flaps in fingertip reconstruction [3].

While these flaps have numerous benefits, they also invariably have some drawbacks, including being technically difficult, usually necessitating a skin graft at the donor site, leaving visible scars, and needing a protracted period of finger immobilization with the consequence of joint stiffness [2].

According to a new Cochrane study, there is insufficient high-level evidence for the fingertip injury treatment, and as a result, it is observed that there is no uniform plan of care between different healthcare practitioners. This acknowledges the requirement for level I data and preventative actions [4].

Electro-photobiomodulation (EPBM), also called E-light, is the process of combining intense pulsed light (IPL) and radiofrequency (RF) to modify tissues to help the body heal itself, lower inflammation, and promote wound healing. EPBM was reported as a safe and effective tool for promoting wound healing in many applications including acute facial burns [5], posttraumatic defects [6], Post-Fournier’s Gangrene peno-scrotal defects [7], hypertrophic scars of hand [8] and face [9], and extensive facial freckles [10].

In this article, we report our results with the utilization of (EPBM) in the treatment of fingertip injuries in comparison with cross finger flap reconstruction.

### Patients and methods

This study was carried out in the Department of Plastic and Reconstructive surgery in our university and private clinic and included 60 patients presented with fingertip injuries. Patients were randomly divided into two groups. Group 1 includes patients who were treated by EPBM. Group 2 includes patients who were treated by cross finger flap (CFF). Approval was obtained from the concerned ethical committee in our university before we commenced this work.

Excluded patients included those with wounds that were not traumatic, wounds that were more than three weeks old, patients on steroid medications, patients with co-morbidities like diabetes or peripheral vascular disease that could affect the survival of the flap, and those with stiffness, arthritis, prior injuries, nerve palsy, and other conditions that could affect the assessment of the results. The study excluded patients who had experienced any extra trauma to the donor finger or those who had any additional injury to soft tissues, bones or joints.

To eliminate debris, devitalized tissues, and foreign bodies, all wounds were debrided and irrigated with saline and povidone-iodine solution.

#### Group 1

Photosensitive patients were excluded. All patients receiving treatment prior to presentation, were not included in the study. The author started E-light treatment sessions (intense pulsed light and radiofrequency) as soon as the patient arrived.

**Fig. 1:**
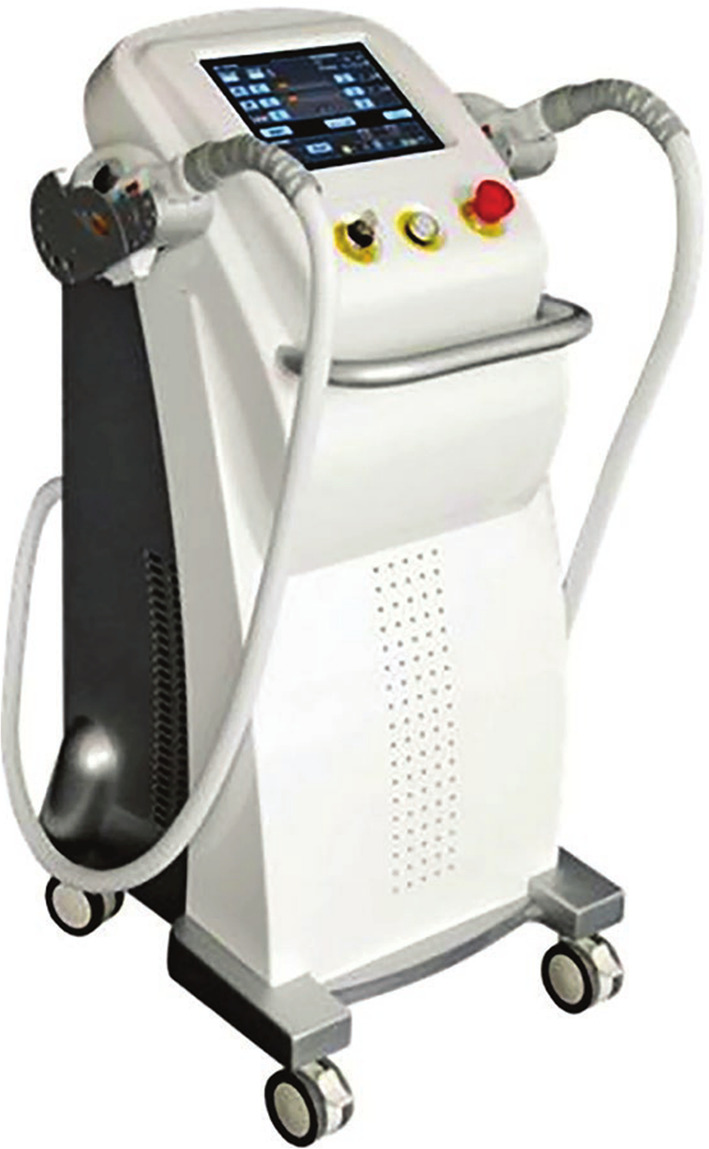
The two-handle E-light beauty machine

The two-handle E-light beauty machine was used in this research. The manufacturer is Beijing Oriental Wison Mechanical & Electronic Co. Ltd. (Fig. 1). Thirty minutes before the E-light session, lidocaine 2.5% gel is applied to the area to be treated.

The fluence of the RF component of E-light was 5–7 joules. The IPL component of E-light had a fluence of 7 to 12 joules, spot diameter of 8 to 32 mm, pulse duration of 3 to 8 milliseconds, pulse delay of 10 to 15 milliseconds, and was combined with different filters according to skin color (560 nm, 580 nm, 630 nm, and 755 nm). Each patient received two sessions per week.

#### Group 2

Local anesthesia, brachial plexus block, or general anesthesia with tourniquet were used for the surgery. Debridement was followed by the standard CFF procedure with skin graft cover of the donor defect. Unaffected digits were mobilized while the wounded digits were splinted. After surgery, the flaps were separated after two to three weeks, and patients began receiving supervised physical therapy that included complete digit mobilization and sensory reeducation. Following surgery, patients were checked on every week for the first six weeks, and then every three, six, nine, and twelve months. Every complication was noted and dealt with appropriately.

Final evaluation of patients of both groups was performed, 6 months after complete healing, and included the following items:

- Occurrence of nail deformity (hook, split, hypertrophic, spike, or absent nails are among the potential deformities).
- Length of injured pulp compared to, and presented as a percent of, contralateral normal.
- Pulp width compared to, and presented as a percent of, contralateral normal.
- Sensory evaluation including:

1- Semmes– Weinstein monofilament (divided into 5 grades starting from grade 1; normal sensation).

2- Static 2-point discrimination (S2PD) (According to Dellon et al., test results were divided into three groups: normal; 1 mm to 5 mm, fair; 6 mm to 10 mm, and bad; 11 mm to 15 mm) [11].

- Using a digital goniometer, the total active range of motion (TAM) of the injured finger was measured. The results were computed and reported as a percentage of the matching contralateral unaffected finger.
- Time to complete healing of injured finger.
- Presence or absence of cold intolerance.
- Patient or parent satisfaction on appearance and function on a score 1–10 where 1 is the lowest satisfaction.
- Independent plastic surgeon evaluation of the result regarding the general aesthetic result of the injured finger and the contour of the cross-finger flap donor site in group 2 patients.
- Adverse events including wound infection, distal necrosis of fingertip, hypertrophic scarring and contracture.

### Statistical analysis

The Statistical Package of Social Sciences (SPSS) for Microsoft Windows version 26 was used to manipulate the data. The mean ± Sd was used to quantify quantitative data, while frequency and percentage were used to measure qualitative data. Using the t test, quantitative data were compared. Chi square is employed to compare qualitative data. In the current investigation, a p-value of less than 0.05 was chosen as the significant level.

## Results (Figs. 2, 3, 4, 5, 6, 7 and 8)

The mean age in group 1 patients was 15.7 years while in group 2 patients it was 17.9 years. There was no statistically significant difference between both groups regarding the age distribution of patients among the different age groups. There was no predilection between either group regarding the gender or the side of hand affected. The thumb was the least affected digit in both groups with no significant difference between both groups regarding the frequency of affected digits. Most patients had Allen 1 or 2 classification of their fingertip injury with no statistically significant difference between both groups. The most common mechanism of injury in both groups was crush injury by door. Demographic and clinical data of both groups are shown in (Table 1).

At the time of final evaluation, the mean pulp length and pulp width were more in group 1 than in group 2, and the difference was statistically significant. Regarding evaluation of sensory recovery using Semmes Weinstein monofilament, group 1 patient had statistically significant much better outcome, with 90% responding to grade 1 filament and the remaining 10% responded to grade 2 filament. On the other hand, near half of group 2 patients responded to grade 1 and 2 filaments and near a third of them responded to grade 4 and 5 filament. Also, sensory recovery measured by S2PD was statistically better in group 1 patients where the majority of them (93.3%) achieved normal sensation. In contrast, only less than half of group 2 patients achieved normal sensation and, their third had poor postoperative sensory recovery as measured by S2PD. Compared to group 2 patients, group 1 patients had statistically significant better TAM, grip strength, patient satisfaction, healing time, and plastic surgeon general aesthetic evaluation and also, they had statistically significant less adverse events and cold intolerance with absent donor site pain and deformity (Table 2).

**Table 1:** Sociodemographic data of patients in both groups

Sociodemographic data	Group I (30 patients)	Group II (30 patients)	*P* value
	Number of patients (%)	Number of patients (%)	
**Age**			
0–10 years	12(40%)	9(30%)	0.7
11–20 years	12(40%)	13(43.3%)	
21–30 years	3(10%)	4(13.3%)	
31–40 years	2(6.7%)	1(3.3%)	
41–50 years	1(3.3%)	3(10%)	
**Sex**:			0.4
male	2(70%)	18(60%)	
Female	9(30%)	12(40%)	
**Hand affected**:			0.1
Right	13(43.3%)	19(63.3%)	
Left	17(16.7%)	11(36.7%)	
**Finger affected**:			0.9
Thumb	2(6.7%)	1(3.3%)	
Index	6(20%)	8(26.7%)	
Middle	11(36.7%)	9(30%)	
Ring	4(13.4%)	6(20%)	
Little	7(23.3%)	6(20%)	
**Allen’s level of injury**:			0.8
I	15(50%)	14(46.7%)	
II	13(43.3%)	11(36.7%)	
III	2(6.7%)	4(13.3%)	
IV	0(0%)	1(3.3%)	
**Mechanism of injury**			7.05 0.07
Crushing by door	23(76.7%)	19(63.3%)	
Crushing by machine	1(3.33)	5(16.7%)	
Crushing by heavy object	0(0%)	3(10%)	
laceration	6(20%)	3(10%)	

**Table 2:** Clinical postoperative evaluation data of patients of both groups

Variable	Group I (30 patients)	Group II (30 patients)	Test of sig
	Number of patients (%)	Number of patients (%)	
Nail deformity	0(0%)	2(6.7%)	0.2
Adequate pulp length	30(100%)	22(73.3%)	0.0001*
Adequate pulp width	30(100%	19(63.3%)	0.0001*
SemmesWeinstein monofilament test:			
1 2 3 4 5	27 (90%) 3(10%) 0(0%) 0(0%) 0(0%)	13(43.3%) 4(13.3%) 3(10%) 5(16.7%) 5(16.7%)	0.001*
S2PD: normal Fair poor	28(93.3%) 2(6.7%) 0	1 (46.7%) 6(20%) 10(33.3%)	0.0001*
TAM as % of contralateral:	100%	77.93%	0.0001*
Grip strength as percent of normal contralateral	100%	86%	t = 4.1, 0.0001*
Healing time	13–28 days	27–42 days	t = 11.4, 0.0001*
Donor site pain	0(0%)	12(40%)	0.0001*
Donor site deformity	0(0%)	27(90%)	0.0001*
Fingertip cold intolerance	6(20%)	21(70%)	0.0001*
Satisfaction: (mean ± SD) -Function -Appearance	9.7 ± 0.5 9.8 ± 0.4	7.6 ± 1.1 5.7 ± 1.4	t = 9.6, 0.0001* t = 15.5, 0.0001*
Plastic surgeon General aesthetic evaluation (mean ± SD)	9.6 ± 0.6	6.5 ± 1.5	t = 10.8, 0.0001*
Adverse events	0(0%)	7(23.3%) (partial flap loss, hypertrophic scar in donor, stiffness)	0.005*

**Fig. 2:**
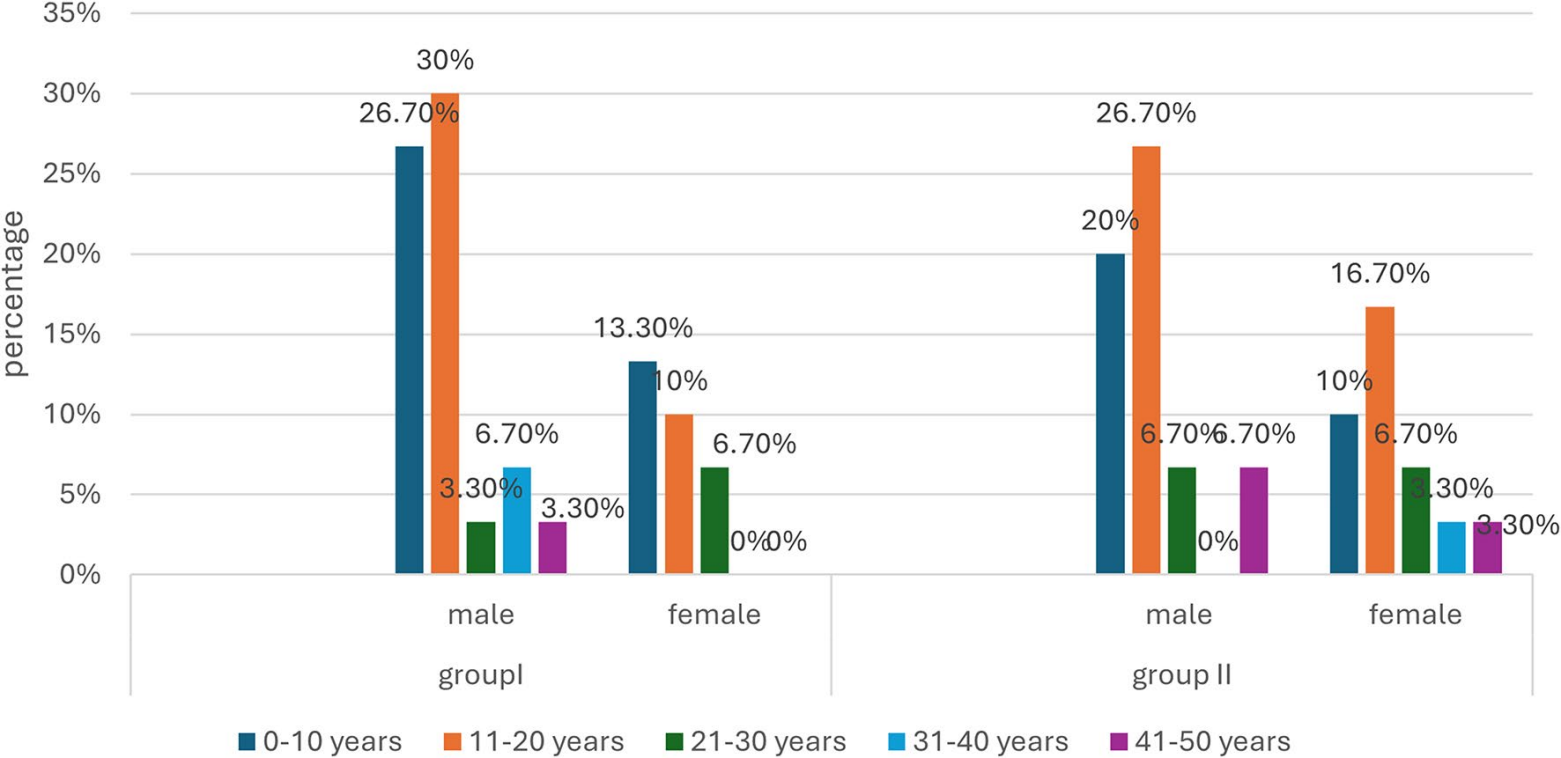
The sex distribution among different age groups of our patients

**Fig. 3:**
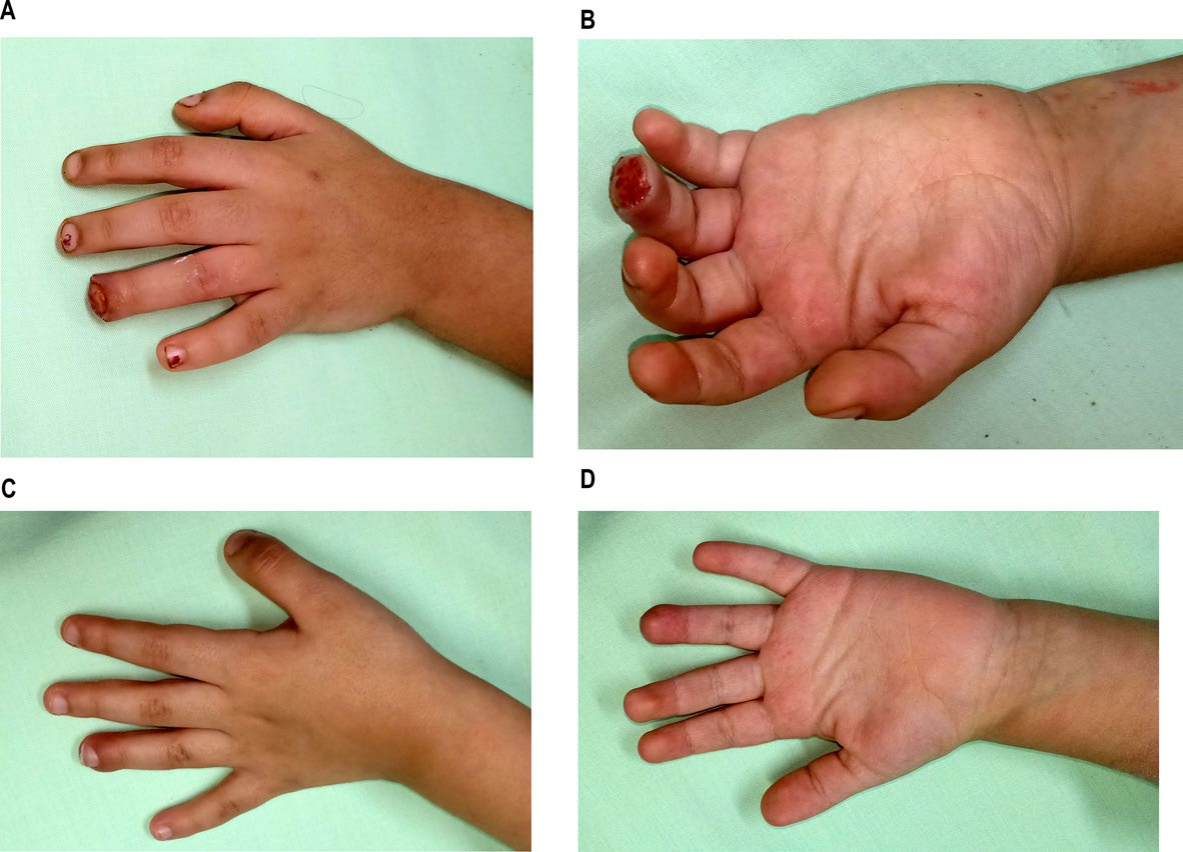
**a**, **b**: ten years old male with fingertip injury before application of EPBM. **c**,**d**: after finishing EPBM sessions

**Fig. 4:**
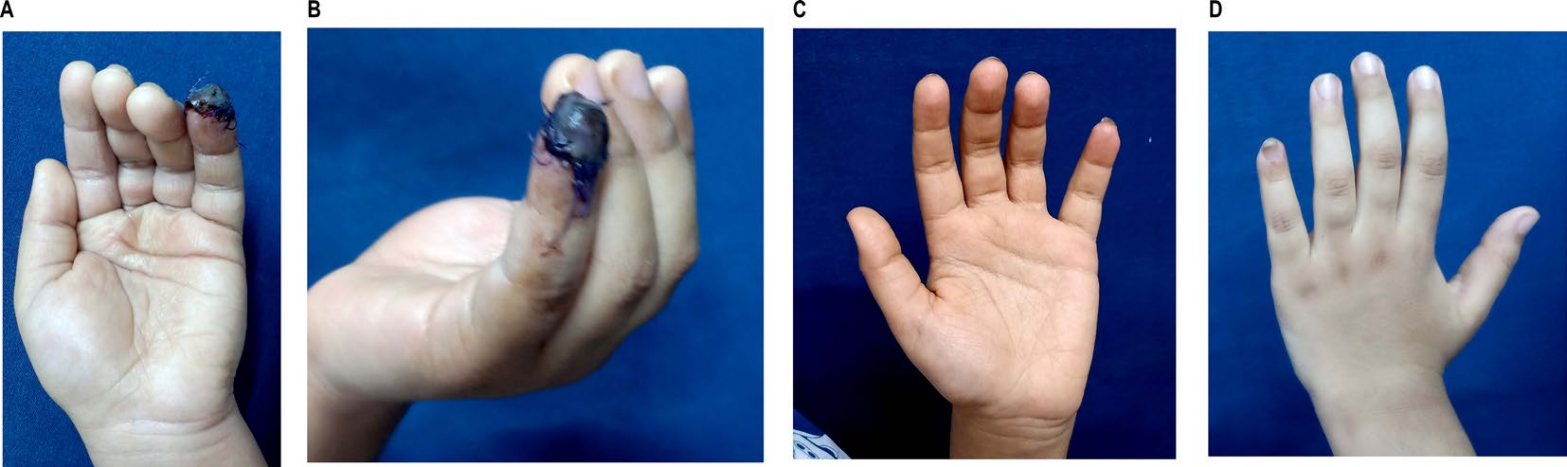
**a**,**b**: eight years old male with fingertip injury before application of EPBM. **c**,**d**: after finishing EPBM sessions

**Fig. 5:**
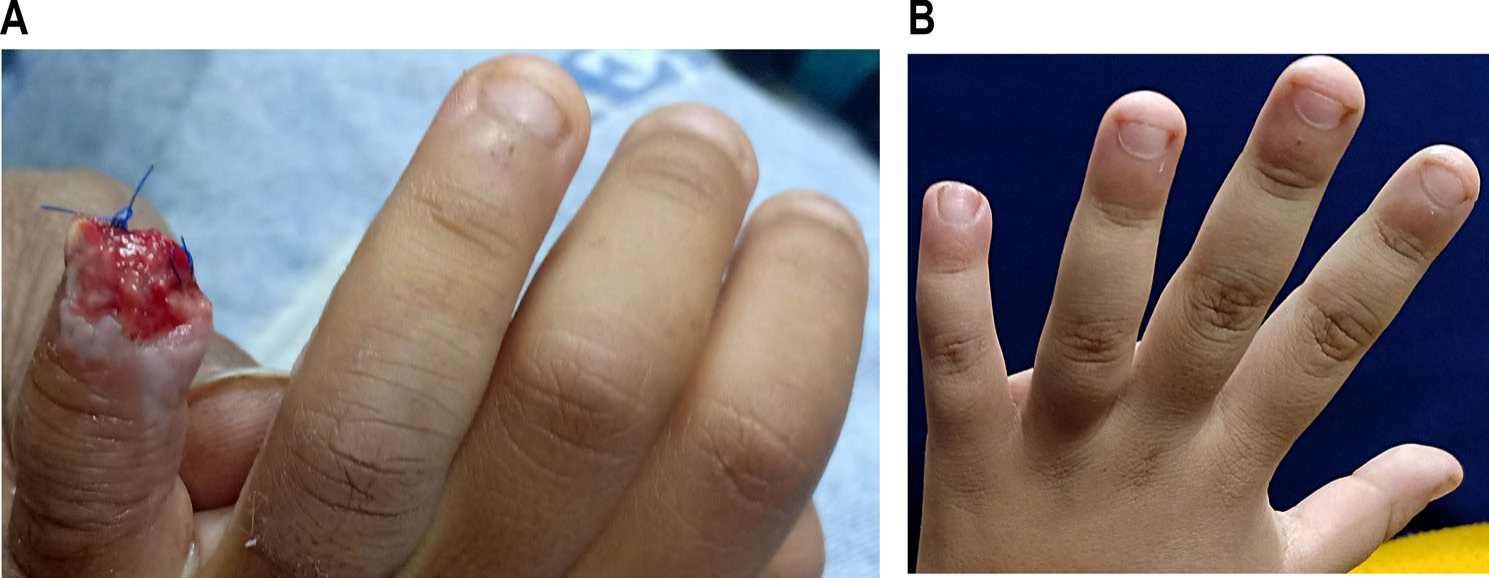
**a**: seven years old male with fingertip injury before application of EPBM. **b**: after finishing EPBM sessions

**Fig. 6:**
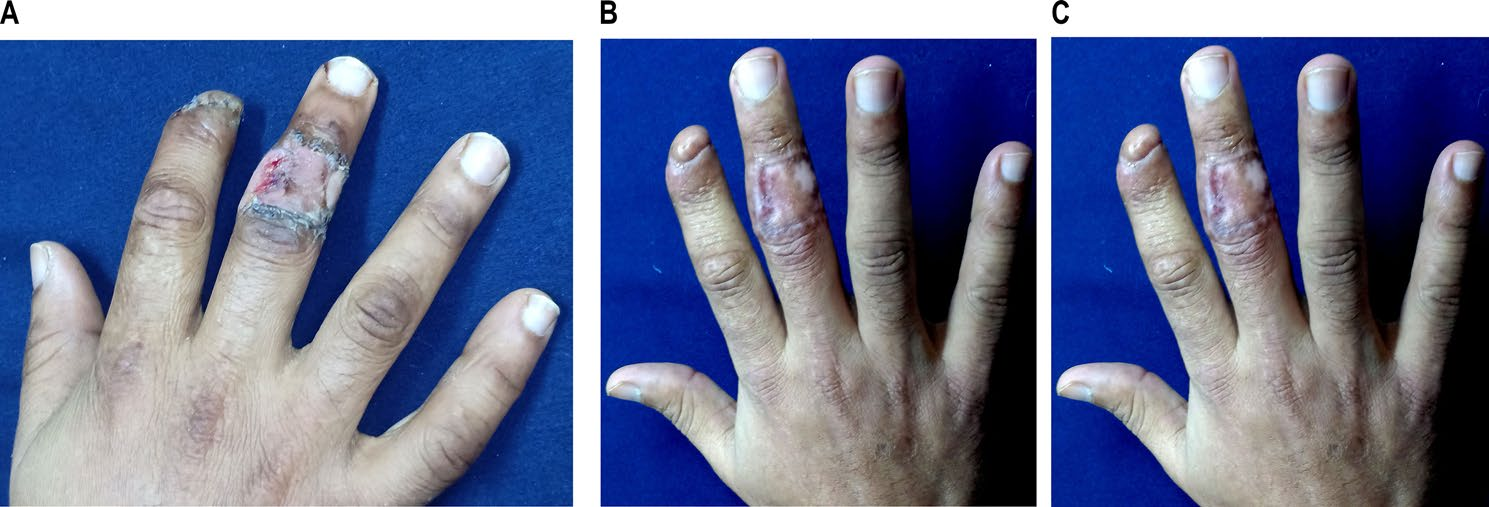
Appearance of donor and recipient site after cross finger flap for cover of fingertip injury: **a**: 2 weeks after flap separation. **b**, **c** three months after flap separation

**Fig. 7:**
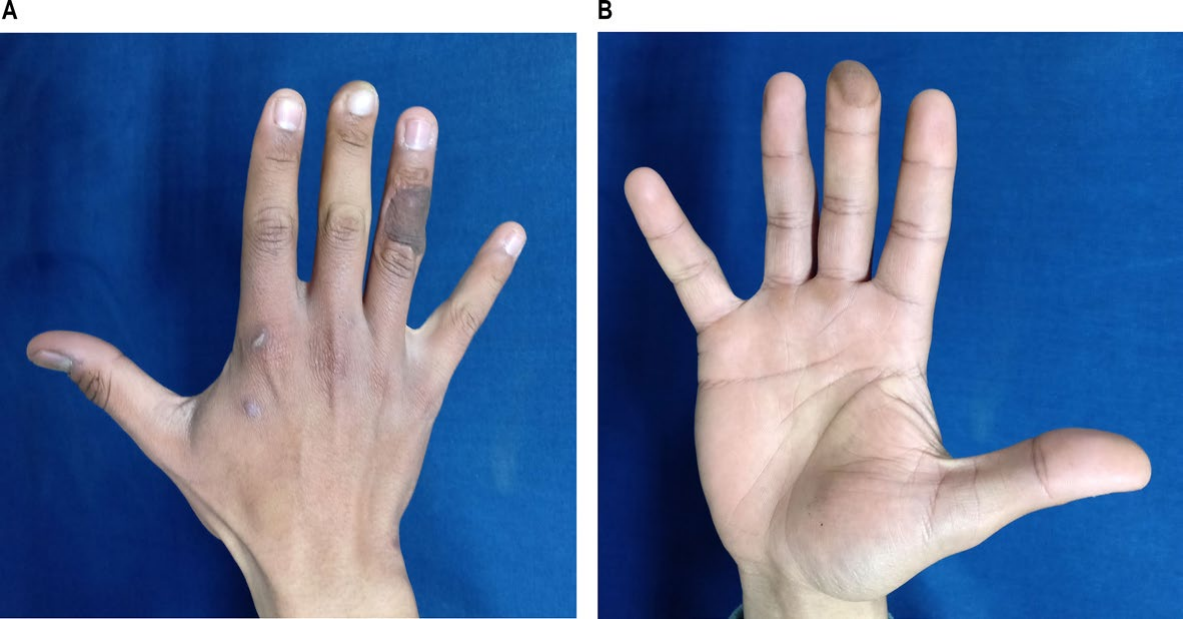
Appearance of donor and recipient site after cross finger flap for cover of fingertip injury three months after flap separation: **a**: posterior view. **b**: anterior view

**Fig. 8:**
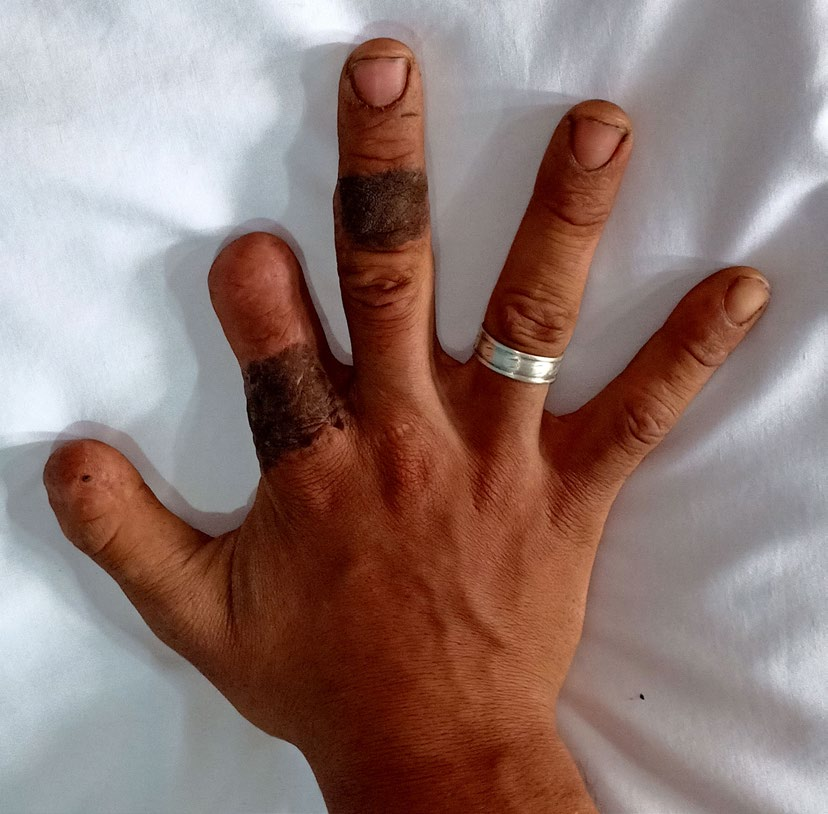
Appearance of donor and recipient site after cross finger flap for cover of fingertip injury three months after flap separation

## Discussion

The fingertip’s unique function and sensory components allow it to serve numerous vital functions in our interactions with the environment. The objectives of treating any damaged fingertip should be to restore a long-lasting, painless sensory interface for manipulating items while maintaining the fingertip’s length, making it appear as normal as possible, and allowing the manipulation of small objects by restoring the nail plate’s geometry [12].

The level of amputation along the wounded finger, the patient’s condition, and the surgeon’s preferences, all influence the treatment plan for fingertip amputations. Treatment options include non-surgical measures (occlusive dressing, medicated gauze dressings) that allow the fingertip to regenerate naturally or covering flaps to replace lost skin. The restoration of fingertip function, including fine sensory perception, durable skin that permits the fingertips to grasp items, and pleasing look, is another goal of this treatment. Coverage flaps have been used to accomplish these objectives over the years. But flaps come with drawbacks like necrosis, infection, diminished sensation, stiff fingers, wound healing consequences, and unstable fingertip [2].

According to one study, the choice of treatment for Allen II, III, and IV fingertip injuries did not affect the result. This is at least a surprising result in an era where the vast array of surgical alternatives suggests that flap reconstruction is optimal [3].

To avoid donor site morbidity, some authors adopted non-surgical measures for treatment of fingertip injuries. However. treatment of fingertip injuries with occlusive dressing resulted in nail dystrophy and ridges especially in Allen zone 3 amputations [13]. 6% of patients treated with moist dressing suffered from a deformity of their nails including hook nails and parrot beak appearances [14]. Additionally, moist dressing treatment resulted in healing time up to 12 weeks in Allen 3 amputations and was associated with cold sensitivity and numbness which negatively impacted their ability to perform everyday tasks [15].

When treating fingertip injuries with exposed bone, other authors utilized semi-occlusive dressings and reported that the average healing period was 6.5 weeks [3–8, 16]. Therefore, compared to alternative reconstructive techniques, this treatment approach is regarded to be time-consuming, may prohibit an early return to work, and may produce a less aesthetically pleasing result [17].

Despite being a simple solution, primary closure of a fingertip lesion causes some loss of digital length and has a reported 13.8% rate of complications, the most prevalent of which are soft-tissue necrosis, painful neuromas, and nail deformities [18]. Skin graft coverage of fingertip injury has many disadvantages including intolerance to cold, hyperesthesia and early loss of sensation [19].

While both homodigital and heterodigital pedicled island flaps can be executed in a single operation, their precise dissection of the neurovascular bundle lengthens the duration of the operation. Furthermore, because reverse flow flaps divide the digital artery, they may result in flap loss [2]. Following the reverse homodigital island flap, cold intolerance developed in up to 100% of patients, which had a detrimental effect on the functional result [20]. These flaps are also linked to certain problems, such as flexion contracture, partial flap necrosis, venous congestion, and cold sensitivity. S2PD on average was 7.2 mm [21]. The most significant adverse effect of reverse homodigital neurovascular island flaps is the presence of postoperative neuropathic pain in 47% of patients, which is most likely caused by compression of the digital nerve following flap rotation to inset into the fingertip defect [22].

Several patients experienced abnormalities in the morphology of their nails and pulp, as well as pain and intolerance to cold in a study evaluating the long-term results of the V-Y advancement flap [23]. When this flap was used on children, 73% of them developed hook nail deformities, and only 43% of them had normal pulp shape [24].

One of the common flaps in thumb fingertip reconstruction is the Moberg flap. However, many studies reported several complications of this flap such as superficial necrosis (17%), infection (17%), and cold intolerance in most patients [14].

The primary disadvantage of these flaps is the restricted amount of advancement that could be made. The Atasoy flap has a limited potential for distal advancement, even though the Moberg flap can cover a 2-cm deficiency of the volar distal thumb. As a result, its application is typically limited to small, 1 cm distal transverse fingertip lesions [19].

The first dorsal metacarpal artery flap, which is taken from an undamaged digit, is another often used flap. A skin graft is needed at the flap donor location. Flap harvest may cause pain or stiffness in the index finger. The skin obtained from the flap is nonglabrous. Because the flap receives innervation from dorsoradial sensory nerve branches, it offers a nonanatomical and poor restoration of sensory function. Reorienting the flap cortically is necessary. Out of all the patients, only half of them identify that the sensation originates from the thumb and not from the dorsum of the index finger [19].

Many studies reported various complications of cross finger flaps. One study reported decreased range of motion (ROM) in 50% of patients, postoperative numbness in 21% of patients, stiffness in 28%, and hyperesthesia in 38%. They noticed that hyperpigmentation of the donor site is the most common complication (82%), and they advised taking into account the possibility of growth restriction due to scarring on the donor digit when selecting this reconstructive option, particularly in younger children who still have a significant amount of longitudinal digital growth left [24].

According to a different study, poor color match and obvious contour deformity were linked to skin graft coverage of the secondary defect in half of the instances (88% hyperpigmented, 12% hypopigmented). There were no clinically significant variations in outcomes between full thickness and split skin grafts for coverage of the donor finger [25]. Other studies reported potential donor finger pain and significantly decreased grip strength and ROM of proximal interphalangeal (PIP) and metacarpophalangeal (MCP) joints after cross finger flap compared to contralateral control [14, 22]. Chitta et al. observed a substantial disparity in pain and appearance between the donor and control digits. They found it unacceptable that 47% of patients had contour deformity and 53.8% showed hyperpigmentation [26].

The findings of our group 2 patients are similar to the above-mentioned studies on cross finger flaps where we had nail deformity in 6.7% of patients, inadequate pulp length in 26.7% and inadequate pulp width in 36.7%, TAM was 77.9% of contralateral normal, grip strength was 86% of contralateral normal, in addition to the donor site pain and deformity which were present in 40% and 90% of group 2 patients respectively. In contrast to this, we find that none of group 1 patients developed nail deformity, donor site pain or deformity or had any degree of deficiency in TAM, grip strength or pulp length or width when compared to the contralateral normal.

The incidence of cold intolerance in cross finger flap patients was reported to be 66% [24], 25% [14], 39% [27], 63% [25], 43% [28], and 32% after average 19.7 years follow up [29]. 70% of our group 2 patients developed cold intolerance compared to only 20% of group 1 patients.

Our group 1 patients had statistically significant better sensory recovery compared to group 2 patients, whether using Semmes-Weinstein monofilament test or S2PD. In a recent systematic analysis of cross finger flaps, the authors found that the postoperative weighted mean two-point discrimination of the donor digits was 8.84 mm, while it was 4.89 mm of contralateral (control) digits. They also found studies reporting significant postoperative reduction in overall range of motion of PIP joints, 32% of patients experienced cold intolerance in the donor digits. 54% had hyperpigmentation, 8% had hypopigmentation, 47% had contour deformity and 10% had pain in the donor digits during follow up period [30].

The thenar flap is known for its major limitations including unsightly donor site and flexion contracture, particularly in individuals who are over the age of thirty [2]. Two stages are needed for the procedure. The resulting skin coverage, inspite of being glabrous, has no sensory perception in the early postoperative period and ends with a mean 7 mm S2PD. The donor site scar usually becomes hypertrophic [19].

When Chakraborty et al. compared the results of thenar flap with CFF, they observed that the former produced greater aesthetic results and sensory recovery. On the other hand, partial flap necrosis was more common in the case of thenar flaps. Additionally, they noticed a statistically significant difference in passive ROM at the PIP and MCP joints between reconstructed and the contralateral normal digit in CFF patients. Nevertheless, this notable distinction in passive ROM between the reconstructed and contralateral normal digit was limited to the PIP joint in the thenar flap group. Cold intolerance occurred in (35%) of thenar flaps and in (43%) of CFFs with no statistically significant difference between both groups [28].

The mean healing time in our study was in group 1 and in group 2. So, EPBM results in a shorter healing time whether compared to surgical or non-surgical treatment options mentioned previously. None of our group 1 patients had adverse events, compared to 23.3% of group 2. Additionally, our group 1 patients had statistically significant better patient satisfaction (with both function and appearance) and plastic surgeon aesthetic evaluation, compared to group 2 patients.

## Limitations

The main limitation in our study may be the limited number of cases however, the results obtained from the present number are valuable and more patients undergoing the same treatment will be reported in further studies to strengthen our findings.

## Conclusion

Based on the findings of the present study, we can conclude that the use of Electrophotobiomodulation is safe and effective treatment of fingertip injuries which shortens the healing time, produces the best aesthetic and functional result while avoiding donor site morbidity of the traditional reconstructive options.

## Data Availability

Authors declare that all data generated or analyzed during this study are included in this published article (and its supplementary information files).

## References

[1] Eberlin KR, Busa K, Bae DS, Waters PM, Labow BI, Taghinia AH. Composite grafting for pediatric fingertip injuries. Hand (New York NY). 2015;10:28–33. 10.1007/s11552-014-9671-5 PMC4349848 25767418

[2] Lemsanni M, Najeb Y, Chaouqui Y, Elkasseh M, Zoukal S. Fingertip injuries managed by a thenar flap: follow-up and long-term outcomes of 32 cases. Hand Surg Rehabilitation. 2021;40:484–90. 10.1016/j.hansur.2021.04.003 33895423

[3] van den Berg WB, Vergeer RA, van der Sluis CK, Ten Duis HJ, Werker PM. Comparison of three types of treatment modalities on the outcome of fingertip injuries. J Trauma Acute care Surg. 2012;72:1681–7. 22695441 10.1097/TA.0b013e318248bc8c

[4] Venkatesh A, Khajuria A, Greig A. Management of Pediatric Distal Fingertip injuries: a systematic literature review. Plast Reconstr Surg Global open. 2020;8:e2595. 10.1097/GOX.0000000000002595 PMC7015615 32095403

[5] Elmelegy NG. Aesthetic treatment of Acute Burns of the Face using Electro-Photobiomodulation. J burn care Research: Official Publication Am Burn Association. 2023;44:1154–61. 10.1093/jbcr/irad009 36708193

[6] Elmelegy NG, Elhawary Y, Sadaka MSJTEJP, Surgery R. Electrophotobiomodulation: A New Step in the Reconstructive Ladder of Post-Traumatic Defects. 2024.

[7] Shoukr T, Elmelegy N, Elghamry S. Isolated penile or scrotal Post-fournier’s gangrene sequelae, the role of electro-photo-biomodulation in conservative treatment. Egypt J Plast Reconstr Surg. 2023;48:1–6.

[8] Elmelegy NG. Hypertrophic scars of the Hand: the role of Electrophotobiomodulation Theory as a recent line of treatment. Plast Reconstr Surg. 2023;151:375–83. 36696322 10.1097/PRS.0000000000009962

[9] Elmelegy NG, Hegazy AM, Sadaka MS, Abdeldaim DE. Electrophotobiomodulation in the treatment of facial post-burn hypertrophic scars in pediatric patients. Annals Burns fire Disasters. 2018;31:127–32. PMC6199016 30374265

[10] Elmelegy N. Epiderm-Abrasion-assisted intensive pulsed light and Radiofrequency in aesthetic treatment extensive facial freckles. Aesthetic Plast Surg. 2020;44:2259–67. 32128707 10.1007/s00266-020-01661-x

[11] Dellon AL, Mackinnon SE, Crosby PM. Reliability of two-point discrimination measurements. J Hand Surg. 1987;12:693–6. 10.1016/s0363-5023(87)80049-7 3655225

[12] Martin-Playa P, Foo A. Approach to Fingertip injuries. Clin Plast Surg. 2019;46:275–83. 31103072 10.1016/j.cps.2019.02.001

[13] Boudard J, Loisel F, El Rifaï S, Feuvrier D, Obert L, Pluvy I. Fingertip amputations treated with occlusive dressings. Hand Surg Rehabilitation. 2019;38:257–61. 10.1016/j.hansur.2019.06.002 31185316

[14] Neustein TM, Payne SH Jr., Seiler JG. 3rd. Treatment of Fingertip injuries. JBJS Reviews. 2020;8:e0182. 32539263 10.2106/JBJS.RVW.19.00182

[15] Masaki S, Kawamoto T. Fingertip Amputation Injury of Allen Type III managed conservatively with moist wound dressings. Am J case Rep. 2021;22:e928950. 33621217 10.12659/AJCR.928950 PMC7913779

[16] Hoigné D, Hug U, Schürch M, Meoli M, von Wartburg U. Semi-occlusive dressing for the treatment of fingertip amputations with exposed bone: quantity and quality of soft-tissue regeneration. J hand Surg Eur Volume. 2014;39:505–9. 10.1177/1753193413489639 23695151

[17] Panattoni JB, De Ona IR, Ahmed MM. Reconstruction of fingertip injuries: surgical tips and avoiding complications. J Hand Surg. 2015;40:1016–24. 10.1016/j.jhsa.2015.02.010 25823622

[18] Harris AP, Goodman AD, Gil JA, Sobel AD, Li NY, Raducha JE, Baird GL, Katarincic JA. Incidence, timing, and risk factors for secondary revision after primary revision of traumatic digit amputations. J Hand Surg. 2018;43:e10401041–10401011. 10.1016/j.jhsa.2018.03.028 29735290

[19] Chang BL, Katz RD. Locoregional options for Acute Volar Pulp Fingertip defects. Hand Clin. 2021;37:11–26. 33198911 10.1016/j.hcl.2020.09.004

[20] Ozaksar K, Toros T, Sügün TS, Bal E, Ademoğlu Y, Kaplan I. Reconstruction of finger pulp defects using homodigital dorsal middle phalangeal neurovascular advancement flap. J hand Surg Eur Volume. 2010;35:125–9. 10.1177/1753193409337957 19687082

[21] Regmi S, Gu JX, Zhang NC, Liu HJ. A systematic review of outcomes and complications of primary Fingertip Reconstruction using Reverse-Flow Homodigital Island flaps. Aesthetic Plast Surg. 2016;40:277–83. 26913519 10.1007/s00266-016-0624-y

[22] Gurbuz K, Dogar F, Yontar Y. Comparison of clinical outcomes of Heterodigital Neurovascular Island Flap, Reverse Homodigital Neurovascular Island Flap, and Cross-finger Flap used for Fingertip Reconstruction. Indian J Orthop. 2022;56:847–55. 35547336 10.1007/s43465-022-00605-8 PMC9043157

[23] Haehnel O, Plancq MC, Deroussen F, Salon A, Gouron R, Klein C. Long-Term Outcomes of Atasoy Flap in Children With Distal Finger Trauma. *The Journal of hand surgery* 2019; 44: 1097.e1091-1097.e1096. 10.1016/j.jhsa.2019.02.018 31005461

[24] Loewenstein SN, Adkinson JM. Pediatric Fingertip injuries. Hand Clin. 2021;37:107–16. 33198910 10.1016/j.hcl.2020.09.009

[25] Paterson P, Titley OG, Nancarrow JD. Donor finger morbidity in cross-finger flaps. Injury. 2000;31:215–8. 10719097 10.1016/s0020-1383(99)00205-3

[26] Chitta M, Malathi L, Joseph A. Cross-finger flap to the Thumb: Quest for an alternate donor. Indian J Plast Surgery: Official Publication Association Plast Surg India. 2020;53:287–92. 10.1055/s-0040-1714181 PMC7458833 32884196

[27] Koch H, Kielnhofer A, Hubmer M, Scharnagl E. Donor site morbidity in cross-finger flaps. Br J Plast Surg. 2005;58:1131–5. 16039623 10.1016/j.bjps.2005.04.047

[28] Chakraborty SS, Dixit PK, Kala PC, Sahu RK, Katrolia D. A prospective trial comparing outcomes at 11 months of a standard Cross-finger Flap versus a laterally based Thenar Flap for Fingertip Reconstruction. J hand Surg Asian-Pacific Volume. 2022;27:49–56. 10.1142/S2424835522500187 35135424

[29] Rabarin F, Saint Cast Y, Jeudy J, Fouque PA, Cesari B, Bigorre N, Petit A, Raimbeau G. Cross-finger flap for reconstruction of fingertip amputations: long-term results. Orthop Traumatol Surg Research: OTSR. 2016;102:S225–228. 27033841 10.1016/j.otsr.2016.03.006

[30] Chakraborty SS, Sahu RK, Acharya S, Goel AD, Midya M, Kotu S. Donor Finger Morbidity in Cross-finger Flap: a systematic review and Meta-analysis. Indian J Plast Surgery: Official Publication Association Plast Surg India. 2023;56:201–7. 10.1055/s-0042-1760092 PMC10332906 37435333

